# Electronic monitoring of adherence to once‐daily and twice‐daily direct oral anticoagulants in patients with atrial fibrillation: Baseline data from the SMAAP‐AF trial

**DOI:** 10.1002/joa3.12532

**Published:** 2021-03-30

**Authors:** Tsuyoshi Shiga, Toshimi Kimura, Noritoshi Fukushima, Yuji Yoshiyama, Kazunori Iwade, Fumiaki Mori, Yoichi Ajiro, Shoji Haruta, Yuichiro Yamada, Emi Sawada, Nobuhisa Hagiwara, the SMAAP‐AF investigators

**Affiliations:** ^1^ Department of Cardiology Tokyo Women’s Medical University Tokyo Japan; ^2^ Department of Clinical Pharmacology and Therapeutics The Jikei University School of Medicine Tokyo Japan; ^3^ Department of Pharmacy Tokyo Women’s Medical University Hospital Tokyo Japan; ^4^ Department of Preventive Medicine and Public Health Tokyo Medical University Tokyo Japan; ^5^ Kitasato University School of Pharmacy Tokyo Japan; ^6^ Department of Cardiology National Hospital Organization Yokohama Medical Center Yokohama Japan; ^7^ Department of Cardiology Tokyo Women’s Medical University Yachiyo Medical Center Yachiyo Japan

**Keywords:** adherence, atrial fibrillation, direct oral anticoagulant, dosing frequency, electronic monitoring

## Abstract

**Background:**

Nonadherence diminishes the efficacy of direct oral anticoagulants (DOACs) in patients with nonvalvular atrial fibrillation (NVAF). This report presents the baseline survey results regarding medication adherence among NVAF patients who were treated with once‐daily edoxaban or twice‐daily apixaban from a randomized control trial of the effect of an educational intervention on DOAC adherence.

**Methods:**

We prospectively studied 301 NVAF patients who were treated with edoxaban (n = 175) or apixaban (n = 126) during the 12‐week observation period. Adherence was measured with an electronic monitoring system and is expressed as the percentage of days with the correct doses in the measurement period (days). Adherence to DOAC therapy was defined based on the standard threshold (≥80%) or a strict threshold (≥90%).

**Results:**

Of the 301 patients, 33 had incomplete data or protocol deviations, leaving 268 patients (edoxaban 158 and apixaban 110) for the per‐protocol baseline analysis. There was no difference in adherence (threshold ≥80%) between the groups (edoxaban 95% vs apixaban 91%, *P* = .2), but there was a lower proportion of patients with strict adherence (threshold ≥90%) among apixaban users than among edoxaban users (edoxaban 87% vs apixaban 76%, *P* = .02). Multivariate analysis showed a negative relationship between apixaban use and an adherence rate ≥90% (odds ratio 0.49, 95% confidence interval [CI]: 0.25‐0.94).

**Conclusions:**

Our study showed that the proportion of DOAC users with adherence (≥80%) did not differ between the groups, but the proportion of patients with strict adherence (≥90%) was lower among those using apixaban than among those using edoxaban.

## Graphical abstract

Electronic monitoring demonstrated that the proportions of NVAF patients with ≥80% adherence did not differ between those taking DOACs once or twice daily in either the full analysis set (FAS) or per‐protocol set (PSS). However, the group taking apixaban twice daily had a lower proportion of patients with ≥90% adherence than the group taking edoxaban once daily.

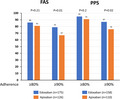

## 1 INTRODUCTION

Atrial fibrillation (AF) is the most common cardiac arrhythmia and has a close relationship with the occurrence of stroke or thromboembolism.^1^, ^2^ For the primary and secondary prevention of stroke, oral anticoagulation with vitamin K antagonists (VKAs) or direct oral anticoagulants (DOACs) are recommended for patients with nonvalvular AF (NVAF).^3^ However, nonadherence to anticoagulation therapy may worsen the prognosis in NVAF patients despite the proven efficacy of anticoagulation therapy for stroke prevention. Unlike VKAs, with DOACs, there is no need to monitor the prothrombin time‐international normalized ratio and/or adjust the dose because of the reduced bleeding risk, and the beneficial effects are also less affected by diet and concomitant medications.^4^, ^5^, ^6^ Therefore, the expectation is that NVAF patients will have better adherence to DOACs than VKAs.

We previously reported that some patients who discontinued DOACs experienced strokes.^7^ We also reported that younger age, frequent daily doses (≥2 times daily), and employment were significantly associated with self‐reported nonadherence to oral anticoagulants, including DOACs.^8^ For NVAF patients treated with DOACs, improving adherence is an important priority. In general, medication adherence decreases as the number of daily doses increases. However, the effect of the dosing frequency of DOACs on adherence remains controversial.

The Survey on Medication Adherence to Anticoagulant Drugs and Investigation of Improvement of Medication Adherence by an Educational Program in Non‐Valvular Atrial Fibrillation (SMAAP‐AF) trial was conducted to examine whether an educational intervention modified patient adherence to DOAC therapy, as measured with an electrical monitoring device. The trial consisted of two periods, namely a 12‐week observation period (Stage 1) and a 12‐week intervention period (Stage 2). In this report, we describe the baseline survey results regarding medication adherence among patients with NVAF who were treated with once‐daily DOACs (edoxaban) or twice‐daily DOACs (apixaban) during the observation period (Stage 1) in the SMAAP‐AF trial.

## 2 METHODS

### 2.1 Study design of the SMAAP‐AF trial

The SMAAP‐AF trial was a multicenter, prospective, interventional study involving patients with NVAF that consisted of two periods: a 12‐week observational period (Stage 1) and a 12‐week single‐blind, randomized, parallel‐group intervention period (Stage 2). In the observation period (Stage 1), medication adherence was investigated among patients with NVAF who were treated with edoxaban once daily or apixaban twice daily. Patients who completed Stage 1 were randomly assigned to the medication educational program or to receive standard medication counseling. Randomization was performed using a minimization method based on medication adherence during Stage 1 and demographic factors as the assignment factors with a 2 × 2 factorial design. Further details on the methods of the SMAAP‐AF trial are provided in the Supplemental Methods (Document S1). This article is a part of the SMAAP‐AF trial and reports the status of medication adherence during the observational period (Stage 1) in patients treated with edoxaban and those treated with apixaban.

Ethics approval was obtained from the institutional review board of Tokyo Women's Medical University and the Certified Review Board of Hattori Clinic, Tokyo, Japan, and written informed consent was obtained from all subjects prior to enrollment. This study was registered with the University Hospital Medical Information Network (UMIN) with the identification number UMIN000031132 and the Japan Registry of Clinical Trials (jRCT) with the number jRCTs031180142.

### 2.2 Patients

This study enrolled outpatients with NVAF who visited the outpatient cardiology clinics of Tokyo Women's Medical University Hospital, the National Hospital Organization Yokohama Medical Center, and Tokyo Women's Medical University Yachiyo Medical Center. The inclusion and exclusion criteria are provided in Table S1. Briefly, study participants were male and female outpatients aged ≥20 years and diagnosed with NVAF who were undergoing treatment with edoxaban or apixaban, with the medication first prescribed at least 4 weeks prior to enrollment in this study. Physicians acted as investigators and research collaborators and recruited the patients. After the eligibility of patients with NVAF was confirmed, those undergoing treatment with either edoxaban or apixaban were enrolled in the trial in the order in which informed consent was obtained.

### 2.3 Assessment of adherence

**FIGURE 1:**
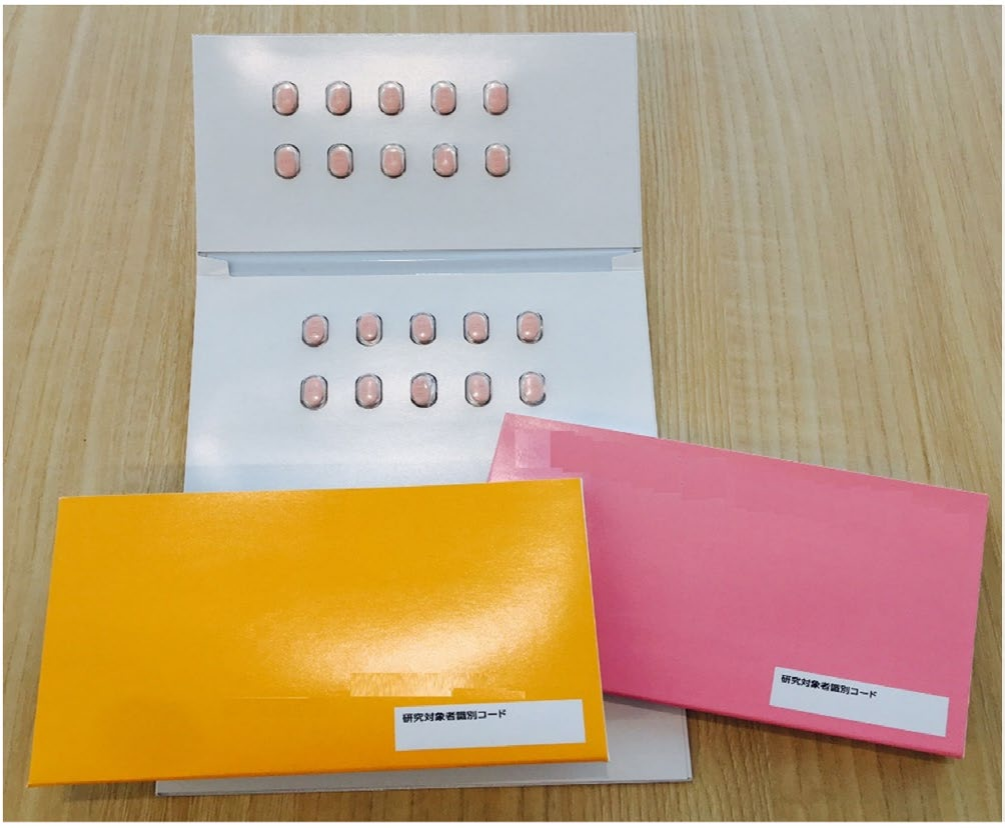
Electronic monitoring device (DNP “Your Manager^®^”). This system is a card‐type press‐through pack (PTP) electronic device that records the date and time when each tablet in the PTP is opened

Medication adherence was measured using the electronic monitoring device “Your Manager^®^” (Dai Nippon Printing Co., Ltd., Tokyo, Japan). “Your Manager^®^” is a card‐type press‐through pack (PTP) electronic device that records the date and time when the packaging for each tablet in the PTP is opened (Figure 1).^9^ Data are recorded in comma‐separated value format.

### 2.4 Data collection

At the time of enrollment, the patients’ demographic information (sex, age, height, weight, employment status, and whether they lived with a partner); cognitive function, as assessed with the Japanese version of the Mini‐Mental State Examination (MMSE)^10^; medical history; concomitant medications including any history of prior anticoagulant use; and Congestive heart failure, Hypertension, Age ≥75 years, Diabetes, Stroke [doubled] (CHADS_2_) score were recorded by the study coordinators. The period for patient enrollment in the SMAAP‐AF study was from March 2018 to November 2018, and the study was completed in May 2019. The patients visited the same pharmacy every time during the study, and the electronic devices were collected by pharmacists during each visit to the pharmacy. All adherence data from the electronic devices were evaluated by independent data assessors but were not made available to patients, physicians, clinic staff, or pharmacists until the last patient completed the trial.

### 2.5 Outcome

Adherence, as measured by the “Your Manager^®^” device, is expressed as the percentage of days with the correct dose in the measurement period for each patient. The variable “days of adherence” was defined as the number of days on which tablets in the PTP were accessed once every 24 hours (between 0300 on a given day and 0300 hours on the next day) for edoxaban or twice daily (one tablet two times daily) every 12 hours (between 0300 and 1500 hours on a given day and between 1500 and 0300 hours on the next day) for apixaban. The variable “days of nonadherence” was defined as no record of accessing a tablet within 24 hours (between 0300 on a given day and 0300 hours on the next day) for edoxaban or no record of accessing a tablet within 12 hours (between 0300 and 1500 hours on a given day or between 1500 and 0300 hours on the next day) for apixaban (missed doses), and as more than one tablet accessed (edoxaban) or more than two tablets accessed (apixaban) within 24 hours (between 0300 and 0300 hours on the next day) (extra doses). We excluded any period during the study in which DOAC therapy was temporarily discontinued by the investigator due to surgery or invasive procedures during the measurement period.

In this study, adherence to DOAC therapy was defined based on (1) the usual threshold, that is ≥80%, which is based on the proportion generally used in the proportion of days covered (PDC) method in the warfarin era^11^, and (2) a strict threshold, that is ≥90%, which was based on the higher adherence rate (PDC ≥90%) associated with the lowest risk in the DOAC era.^12^

### 2.6 Statistics

Summary data are presented as the number of patients (percentage) or the median and interquartile range. Creatinine clearance was calculated using the Cockcroft‐Gault formula.^13^ Comparisons between groups were performed using Student's *t* test for normally distributed continuous variables (with normality assessed with the Shapiro‐Wilk test) or the Mann‐Whitney U test for other variables. Categorical variables were analyzed with the Chi‐square test.

To determine the variables predictive of DOAC adherence, univariate and multivariate logistic regression models adjusted for demographic variables were constructed using edoxaban use/apixaban use and the following baseline characteristics: sex, age, CHADS_2_ score, stroke‐related comorbidities, antiplatelet/nonsteroidal anti‐inflammatory drug (NSAID) use, MMSE score ≤23, living alone, and active employment. The results of the analyses were described as odds ratios (ORs) and 95% confidence intervals (95% CI) for favorable DOAC adherence according to both thresholds (≥80% and ≥90%). A variable with an OR >1 was considered a significant factor associated with favorable DOAC adherence, while a variable with an OR <1 was considered to be associated with nonadherence. Data analyses were performed with IBM SPSS Statistics version 26 (IBM Japan, Tokyo, Japan).

The patients included in the analysis were handled as follows: the full analysis set (FAS) comprised all patients who received at least one dose of the study DOACs in the observation run‐in period and had sufficient records from the electronic monitoring device. The per‐protocol set (PPS), a subset of the FAS, included patients who received consistent treatment with the same DOAC throughout the study period and whose data were recorded with the electronic monitoring device for 60 days or more. Patients whose total discontinuation period was longer than 30 days were removed from the PPS analysis. Patients with incomplete electronic records were also removed from the PPS analysis. The analysis of the outcome was performed with the PPS and was repeated with the FAS to assess the robustness of the results. In the FAS, missing data were imputed as “not taking the same DOAC”.

## 3 RESULTS

**FIGURE 2:**
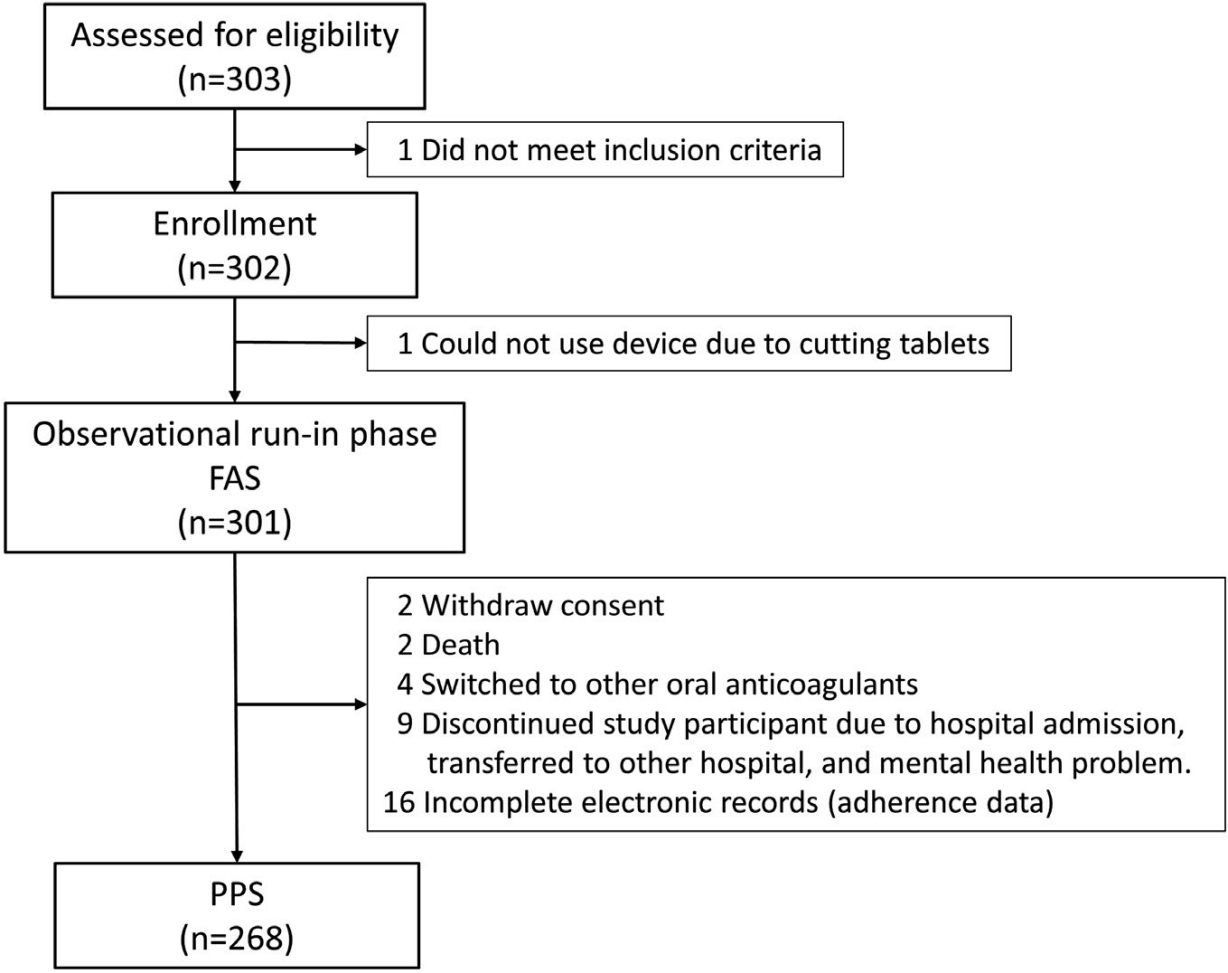
Patient disposition. FAS, full analysis set; PPS, per‐protocol set

Of the 301 patients who entered the observation run‐in phase (mean age of 73 years, 37% women, 36% employed, and 20% living alone), 63% had hypertension, 41% had diabetes, 42% had heart failure, and 12% had had a prior stroke/transient ischemic attack (TIA). The median CHADS_2_ score was 2. Among these patients, 175 (58%) were treated with edoxaban, and 126 (42%) were treated with apixaban at the time of enrollment in this study. Of these patients, 33 had incomplete data or protocol deviations, leaving 268 subjects for inclusion in the PPS (Figure 2).

The baseline characteristics of the patients in the FAS and PPS who were treated with edoxaban and with apixaban are shown in Table 1. The proportion of patients who had had a stroke/TIA and the proportion of patients using antiplatelets were higher in the group treated with apixaban than in the group treated with edoxaban. Creatinine clearance tended to be lower in the group receiving apixaban than in the group receiving edoxaban. The proportion taking a reduced dose was 65% in the edoxaban group and 44% in the apixaban group. Among the study subjects, 102 (58%) of the 175 patients treated with edoxaban and 31 (25%) of the 126 patients treated with apixaban met the criteria for dose reduction. However, there was no significant difference in age, sex, CHADS_2_ score, MMSE, partner status, or work status between the groups.

**TABLE 1:** Baseline characteristics

	FAS			PPS		
	Edoxaban	Apixaban	*P* value	Edoxaban	Apixaban	*P* value
	(n = 175)	(n = 126)		(n = 158)	(n = 110)	
Male sex	108 (62)	81 (64)	.65	97 (61)	67 (61)	.94
Age, years	76 (69‐80)	76 (68‐81)	.93	75 (69‐79)	76 (66‐80)	.97
Body weight, kg	60 (52‐69)	62 (53‐68)	.97	61 (53‐70)	62 (53‐69)	.98
Creatinine clearance, mL/min	59 (44‐72)	55 (39‐68)	.03	61 (45‐73)	56 (41‐69)	.09
CHADS_2_ score	2 (1‐3)	2 (1‐3)	.75	2 (1‐3)	2 (1‐3)	.53
Stroke‐related comorbidities						
Heart failure	81 (46)	51 (40)	.32	70 (44)	43 (39)	.4
Coronary artery disease	28 (16)	17 (13)	.55	23 (15)	16 (15)	.99
Hypertension	109 (62)	86 (68)	.28	94 (60)	75 (68)	.15
Diabetes mellitus	76 (43)	48 (38)	.35	69 (44)	40 (36)	.23
Previous stroke/TIA	14 (8)	24 (19)	<.01	11 (7)	20 (18)	<.01
Peripheral artery disease	16 (9)	16 (17)	.32	16 (10)	13 (12)	.66
Antiplatelet use	6 (3)	13 (10)	<.01	4 (3)	9 (8)	.03
NSAID use	3 (2)	3 (2)	.68	3 (2)	3 (3)	.65
MMSE			.18			
≤23	1 (1)	3 (2)		0	0	
>23	174 (99)	123 (98)		158 (100)	110 (100)	
Living status						
Living alone	33 (19)	26 (21)	.7	31 (20)	23 (21)	.8
Work status						
Employed	62 (35)	47 (37)	.74	59 (38)	42 (38)	.89
Daily dose of DOAC			<.01			<.01
Standard dose	61 (35)	71 (56)		57 (36)	63 (57)	
Reduced dose*	114 (65)	55 (44)		101 (64)	47 (43)	

Note

Values are n (%) or median [interquartile range].

FAS, full analysis set; NSAID, nonsteroidal anti‐inflammatory drug; MMSE, Mini‐Mental State Examination; PPS, per‐protocol set; TIA, transient ischemic attack. CHADS_2_ = cardiac failure, hypertension, age ≥ 75 years, diabetes, previous stroke or TIA (doubled).

* Reduced doses were edoxaban 30 mg once daily and apixaban 2.5 mg twice a daily.

**FIGURE 3:**
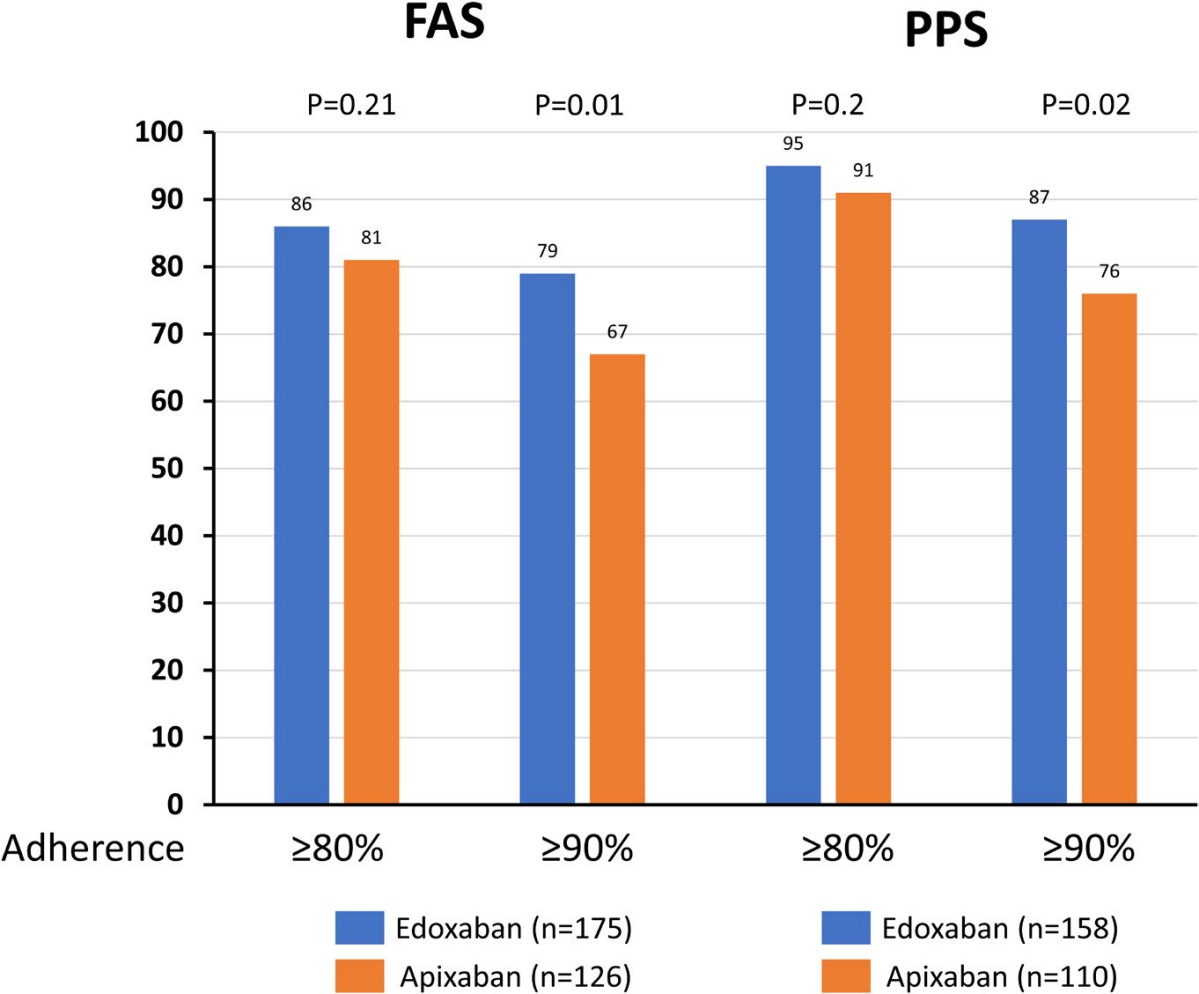
Adherence to study direct oral anticoagulants. FAS, full analysis set; PPS, per‐protocol set

The proportion of DOAC users meeting the adherence threshold of ≥80% did not differ between the groups, but the proportion of DOAC users meeting the strict threshold of ≥90% was lower in the group treated with apixaban than in the group treated with edoxaban (Figure 3).

In the multivariate analysis, there were no consistent relationships between demographic/medical variables and adherence (≥80%) in either the FAS or PSS (Table 2). Advanced age and antiplatelet/NSAID use tended to be associated with nonadherence in the FAS, but these relationships disappeared in the PPS. On the other hand, female sex was associated with nonadherence to the PPS despite the absence of any difference between males and females in the FAS. Among 48 nonadherent patients in the FAS, 22 (80%) of the 27 male patients and 8 (39%) of the 21 female patients were excluded from the PPS. The different results between the FAS and PPS were due to this difference in withdrawal rate. However, there was a negative relationship between apixaban use and strict adherence (≥90%) in both the FAS and PSS (odds ratio = 0.56, 95% CI: 0.32‐0.91 and odds ratio = 0.49, 95% CI: 0.25‐0.94, respectively) (Table 3). Other variables were not found to be associated with strict adherence to the FAS or PPS.

**TABLE 2:** Odds ratio for adherence ≥80%

	FAS				PPS			
	Univariate analysis		Multivariate analysis		Univariate analysis		Multivariate analysis	
	OR (95% CI)	*P* value	OR (95% CI )	*P* value	OR (95% CI )	*P* value	OR (95% CI )	*P* value
DOAC								
Edoxaban	1		1		1		1	
Apixaban	0.68 (0.36‐1.26)	.21	0.72 (0.38‐1.37)	.32	0.53 (0.20‐1.40)	.2	0.58 (0.21‐1.63)	.3
Sex								
Male	1		1		1		1	
Female	0.72 (0.39‐1.35)	.31	0.73 (0.37‐1.44)	.36	0.22 (0.08‐0.64)	<.01	0.19 (0.06‐0.59)	<.01
Age								
1 year increase	0.95 (0.92‐0.99)	.01	0.96 (0.91‐0.99)	.04	0.99 (0.94‐1.04)	.61	0.99 (0.93‐1.06)	.81
CHADS_2_ score								
1 unit increase	0.87 (0.71‐1.07)	.18	1.04 (0.78‐1.37)	.8	1.00 (0.71‐1.39)	.98	1.09 (0.22‐5.43)	.92
Presence of stroke‐related comorbidities*								
Yes	1.43 (0.57‐3.58)	.44	1.22 (0.42‐3.59)	.71	1.12 (0.31‐4.04)	.87	0.26 (0.06‐1.17)	.08
No	1		1		1		1	
Antiplatelet/NSAID use								
Yes	0.36 (0.15‐0.89)	.03	0.41 (0.16‐1.07)	.07	0.34 (0.09‐1.30)	.17	1.10 (0.71‐1.73)	.66
No	1		1		1		1	
MMSE score								
≤23	0.56 (0.06‐5.54)	.62	0.81 (0.08‐8.57)	.86	NA (No patients with an MMSE scores ≤ 23)			
>23	1		1		1		1	
Living status								
Alone	1		1		1			
With others	1.10 (0.51‐2.35)	.82	0.87 (0.39‐1.97)	.87	1.58 (0.54‐4.64)	.41	1.10 (0.34‐3.56)	.88
Working status								
Yes	1.64 (0.83‐3.28)	.15	1.03 (0.47‐2.24)	.94	1.23 (0.45‐3.38)	.69	0.74 (0.23‐2.40)	.61
No	1		1		1		1	

Note

CI, Confidence Interval; DOACs, direct oral anticoagulants; FAS, full analysis set; MMSE, Mini‐Mental State Examination; NA, not applicable; NSAID, nonsteroidal anti‐inflammatory drug; OR, odds ratio; PPS, per‐protocol set.

CHADS_2_ = cardiac failure, hypertension, age ≥ 75 years, diabetes, previous stroke or transient ischemic attack (doubled).

* Stroke‐related comorbidities: heart failure, coronary artery disease, hypertension, diabetes mellitus, previous stroke/transient ischemic attack, and peripheral artery disease.

**TABLE 3:** Odds ratio for adherence ≥ 90%

	FAS				PPS			
	Univariate analysis		Multivariate analysis		Univariate analysis		Multivariate analysis	
	OR (95% CI )	*P* value	OR (95% CI )	*P* value	OR (95% CI )	*P* value	OR (95% CI )	*P* value
DOACs								
Edoxaban	1		1		1		1	
Apixaban	0.52 (0.31‐0.87)	.01	0.56 (0.33‐0.96)	.03	0.47 (0.25‐0.89)	.02	0.49 (0.25‐0.94)	.03
Sex								
Male	1		1		1		1	
Female	0.93 (0.55‐1.58)	.79	0.90 (0.51‐1.60)	.73	0.64 (0.34‐1.21)	.17	0.62 (0.31‐1.24)	.18
Age								
1 year increase	0.97 (0.94‐0.99)	.04	0.98 (0.94‐1.01)	.2	0.99 (0.96‐1.03)	.65	0.99 (0.95‐1.04)	.76
CHADS_2_ score								
1 unit increase	0.87 (0.73‐1.03)	.11	0.94 (0.50‐1.97)	.99	0.95 (0.77‐1.19)	.67	1.13 (0.39‐3.27)	.83
Presence of stroke‐related comorbidities*								
Yes	1.09 (0.54‐2.22)	.8	0.81 (0.34‐1.89)	.62	0.99 (0.41‐2.40)	.99	0.59 (0.19‐1.84)	.37
No	1		1		1		1	
Antiplatelet/NSAID use								
Yes	0.41 (0.18‐0.94)	.04	0.52 (0.22‐1.26)	.15	0.55 (0.19‐1.62)	.28	0.99 (0.75‐1.31)	.94
No	1		1		1		1	
MMSE score								
≤23	0.11 (0.01‐1.10)	.06	0.16 (0.02‐1.65)	.12	NA (No patients with an MMSE scores ≤ 23)			
>23	1		1		1		1	
Living status								
Alone	1		1		1		1	
With others	1.08 (0.57‐2.05)	.81	0.99 (0.50‐1.97)	.99	1.31 (0.62‐2.78)	.49	1.15 (0.52‐2.56)	.74
Working status								
Yes	1.39 (0.80‐2.41)	.25	1.07 (0.56‐2.05)	.84	1.04 (0.54‐2.00)	.91	0.84 (0.39‐1.83)	.67
No	1		1		1		1	

Note

CI, confidence interval; CV, cardiovascular; DOACs, direct oral anticoagulants; FAS, full analysis set; MMSE, Mini‐Mental State Examination; NA, not applicable; NSAID, nonsteroidal anti‐inflammatory drug; OR, odds ratio; PPS, per‐protocol set.

CHADS_2_ = cardiac failure, hypertension, age ≥ 75 years, diabetes, previous stroke or transient ischemic attack (doubled).

* Stroke‐related comorbidities: heart failure, coronary artery disease, hypertension, diabetes mellitus, previous stroke/transient ischemic attack, and peripheral artery disease.

There were nine serious adverse events among the 301 patients during the observation period (Table 4). No stroke/systemic embolism or major bleeding events were observed in patients who were treated with edoxaban or apixaban. Three (1%) patients had serious adverse events leading to discontinuation (two patients died, and one patient experienced severe low back pain). The other patients continued the same DOAC therapy during the observation period.

**TABLE 4:** Serious adverse events

	Edoxaban	Apixaban
Death		
Sudden death	1	0
Pneumonia	0	1
Hospitalization		
Heart failure	0	1
Cather ablation for AF	1	1
Severe low back pain	1	0
Hyperparathyroidism	1	0
Urinary tract infection	0	1
Cataract surgery	0	1

Note

AF, atrial fibrillation

## 4 DISCUSSION

This study involved 12 weeks of electronic monitoring of treatment adherence in Japanese patients with NVAF and showed the following results: 1) the proportion of patients who adhered to treatment according to the usual threshold (≥80%) did not differ between the group taking once‐daily edoxaban and the group taking twice‐daily apixaban, 2) the proportion of patients who adhered to treatment using the strict threshold (≥90%) was lower in the group taking twice‐daily apixaban than in the group taking once‐daily edoxaban, and 3) multivariate analysis showed a negative interaction between the use of twice‐daily apixaban and adherence (≥90%).

There are many reports on adherence to treatment with oral anticoagulants. There are various adherence measurement methods, such as the medication possession ratio (the ratio of the total number of days that the target drug should be taken to the total number of days the medication was actually taken as assessed with the remaining drug survey: MPR), the PDC (the ratio of the total number of days covered to the number of days in the target period), medication event monitoring system (automatic medication recording vial: MEMS), and self‐report.^14^ Generally, a PDC ≥80% is considered adherence.^12^ For warfarin, a previous study reported that patients who missed more than 20% of the doses (1‐2 missed days each week) had more than a twofold higher odds of underanticoagulation.^15^ In the era of DOACs, a recent cohort study using the Korean National Health Insurance Service database showed that the lack of adherence to DOAC therapy (PDC <80%) led to the failure to achieve a better efficacy with regard to preventing ischemic stroke/systemic embolism compared to the use of warfarin, and that maintaining ≥90% adherence optimizes the effectiveness of DOAC therapy.^13^

Although the clinical benefits of DOACs have been shown in many studies, nonadherence diminishes the efficacy of DOACs.^16^ Although the methods of measuring adherence were different, the results of several cohort studies suggested that the rate of adherence to DOAC therapy was 39%–99%.^17^, ^18^, ^19^, ^20^, ^21^, ^22^, ^23^, ^24^, ^25^, ^26^ These data indicate that the rate of nonadherence (threshold <80%) is approximately 25% to 50% in patients taking DOACs.

A systematic review described that medication adherence decreases as the number of daily doses increases.^27^ A meta‐analysis of the effect of the dosing frequency of chronic cardiovascular drugs on adherence also showed that adherence was greater to once‐daily doses than to more frequent (two to four times) daily doses.^28^ Several cohort studies have investigated this issue in DOACs, but their results were not consistent. US and German cohort studies showed that adherence to once‐daily rivaroxaban was greater than adherence to twice‐daily dabigatran or apixaban.^29^, ^30^, ^31^ In our study, among the 126 patients taking twice‐daily apixaban, more patients missed evening doses (between 1500 and 0300 hours on the next day, n = 56) than morning doses (between 0300 and 1500 hours, n = 32) or both evening and morning doses (n = 15). A recent small study using electronic monitoring reported that stroke patients treated with twice‐daily DOACs had lower adherence to evening doses than to morning doses^32^, and missed evening doses may be common among patients treated with twice‐daily DOACs.

However, large cohort studies reported that adherence to twice‐daily apixaban was greater than adherence to rivaroxaban or dabigatran.^26^, ^33^ A Korean cohort study reported that twice‐daily dosing was associated with better adherence.^13^ Adherence to DOACs might be affected by factors beyond simply the number of doses per day.^34^ Until now, no real‐world data regarding adherence to edoxaban have been available. This study is the first to report adherence to edoxaban and compare the new once‐daily regimen for edoxaban with the twice‐daily regimen for apixaban, which has been shown to have adequate adherence.

Dunbar‐Jacob et al suggested that differences in the methods used to measure adherence, such as electronic monitoring, PDC, and self‐report, contribute to differences in the identified predictors of adherence.^35^ To ensure the accurate measurement of adherence to medication regimens, electronic monitoring is preferred and may be considered the gold standard.^36^ In our study, which involved the use of an electronic monitoring device, during the observation run‐in period, the proportion of patients adhering to the treatment regimen according to the strict threshold (≥90%) was higher in the group receiving once‐daily edoxaban than in the group receiving twice‐daily apixaban, although when the usual threshold (≥80%) was applied, there was no difference in adherence between the two groups. When an adherence threshold >80% was applied, adherence to DOACs was superior to adherence to warfarin.^26^ In the DOAC era, however, a strict adherence threshold >90% will be needed to ensure the superiority of DOACs to warfarin with regard to effectiveness. In the future, specific education will need to be provided to nonadherent patients taking DOACs to improve their adherence. For patients taking warfarin, educational interventions have been reported to significantly improve anticoagulation control (time within the therapeutic range) in patients with NVAF.^37^ In the 12‐week randomized intervention period of the SMAAP‐AF trial, we evaluated whether an educational intervention involving motivational interviewing would modify patient adherence to treatment with DOACs.

### 4.1 Study limitations

This study has several limitations. The selection of DOACs was not randomized during the observation period. The proportion of patients who had a prior stroke/TIA and the proportion of patients who used antiplatelets were higher in the group receiving apixaban than in the group receiving edoxaban. Creatinine clearance tended to be lower in the group receiving apixaban than in the group receiving edoxaban. These selection biases might be partially related to physician and patient preferences, associated comorbidities, and concomitant medications. The proportion of patients taking a reduced dose was higher in the group treated with edoxaban than in the group treated with apixaban. Although off‐label users of reduced doses were partially included, the characteristics of the study subjects who had to consider taking a reduced dose (eg, old age, low body weight, and low creatinine clearance) were indications for the selection of a reduced dose. We used well‐known individual and sociodemographic variables associated with medication adherence to adjust the model, but residual unmeasured confounding may have remained. We could not completely exclude the influences of several confounding clinical and health behavior factors on adherence. The numbers of patients taking edoxaban and apixaban were not equal. Since this study was a short‐term evaluation with a small sample size, the effect of adherence on the clinical outcome could not be evaluated. In this study, there were no strokes/systemic embolisms, major bleeding events, or adverse events related to DOAC use during the observation period.

## 5 CONCLUSIONS

Our study involved the use of an electronic monitoring device and showed that the proportion of DOAC users meeting the adherence threshold of ≥80% did not differ between the group treated with once‐daily edoxaban and twice‐daily apixaban, but the proportion of patients who adhered to treatment using the strict threshold of ≥90% was lower in the group treated with apixaban than in the group treated with edoxaban during an observational run‐in period. In the DOAC era, specific education for nonadherent patients will be needed to maintain ≥90% adherence, which is the threshold that must be met to optimize the effectiveness of DOAC therapy.

## CONFLICT OF INTEREST

T Shiga and N Hagiwara received lecture fees from Daiichi Sankyo and Bristol‐Myers Squibb. N Hagiwara received research funding from Daiichi Sankyo. The other authors declare that they have no competing interests.

## Supporting information

Doc S1

Table S1

## APPENDIX A

### SMAAP‐AF INVESTIGATORS

*Principal Investigator:* Tsuyoshi Shiga (Tokyo Women's Medical University, The Jikei University School of Medicine); *Project Manager:* Emi Sawada (Tokyo Women's Medical University); *Institutional Chief Investigators:* Kazunori Iwade (National Hospital Organization Yokohama Medical Center), Shoji Haruta (Tokyo Women's Medical University Yachiyo Medical Center), Nobuhisa Hagiwara (Tokyo Women's Medical University Hospital); *Investigators:* Fumiaki Mori, Yoichi Ajiro (National Hospital Organization Yokohama Medical Center), Yuichiro Yamada (Tokyo Women's Medical University Yachiyo Medical Center); *Safety Assessment:* Yoshio Uetsuka (Institute of Geriatrics, Tokyo Women's Medical University); *Statistical Analysis:* Noritoshi Fukushima (Tokyo Medical University); *Patient Education Advisors:* Yuji Yoshiyama (Kitasato University School of Pharmacy) and Toshimi Kimura (Tokyo Women's Medical University Hospital).
