# Supplementary material for: Electronic monitoring of adherence to once‐daily and twice‐daily direct oral anticoagulants in patients with atrial fibrillation: Baseline data from the SMAAP‐AF trial
Source: J Arrhythm. 2021 Mar 30;37(3):616–25. doi: 10.1002/joa3.12532 (PMC8207342; doi:10.1002/joa3.12532)
Supplement: Supplementary file 1 — Doc S1 [file JOA3-37-616-s001.docx]

**Supplemental Methods**

**Patients**

This study enrolled outpatients with NVAF who visited the outpatient cardiology clinics of Tokyo Women's Medical University Hospital, National Hospital Organization Yokohama Medical Center and Tokyo Women's Medical University Yachiyo Medical Center. The inclusion and exclusion criteria are provided in Table S1. Briefly, study patients will consist of male and female outpatients aged ≥20 years diagnosed with NVAF who were undergoing treatment with edoxaban or apixaban, with the medication first prescribed at least 4 weeks prior to enrollment in this study. Physicians as investigators and research collaborators will recruit patients. After confirming the eligibility of patients with NVAF, those undergoing treatment with either edoxaban or apixaban will be enrolled for Stage 1 in the order in which informed consent is obtained.

**Study design**

The SMAAP-AF study is a multicenter, prospective, interventional study of patients with NVAF and consists of two periods, namely, an observational period of 12 weeks (Stage 1) and a 12-week single-blind, randomized, parallel-group intervention (Stage 2) (Figure S1). In Stage 1, the status of medication adherence will be investigated among patients with NVAF being treated with edoxaban once daily or apixaban twice daily. Patients who complete the Stage 1 observation will be randomized 1:1 using a minimization method based on medication adherence in Stage 1 and demographic items as an assignment factor with a 2 × 2 factorial design randomizing an equal number of patients to receive the medication educational program or to receive standard medication counseling using a Web-based dynamic random allocation system (Figure).


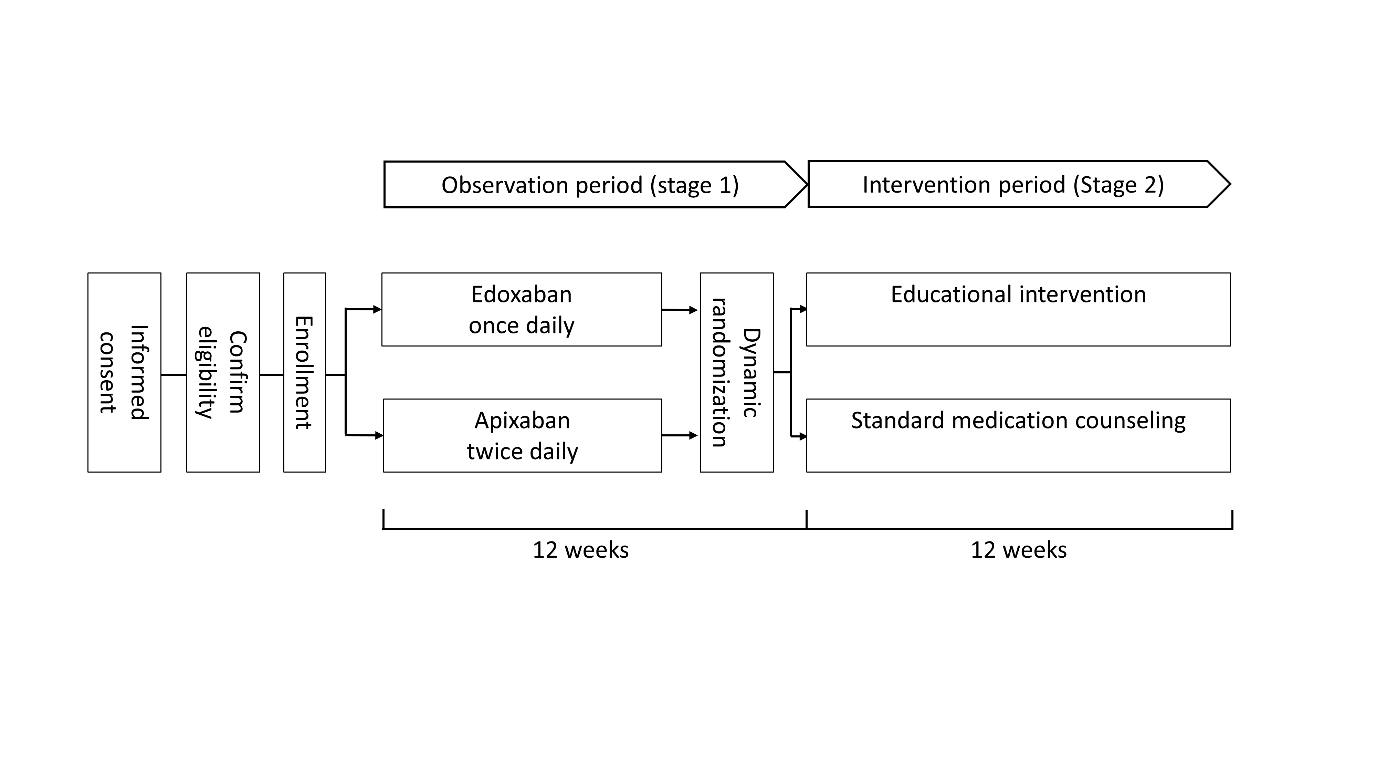


Figure. Schematic representation of the study design

The hospital pharmacists or health insurance pharmacy pharmacists as research collaborators will assign participants to intervention according to. Therefore, physicians, clinic staff, outcome assessors and data analysts will be blinded to the intervention arm the patients is randomized to. In Stage 2, changes in medication adherence in patients with NVAF will be compared between those who received an educational program for drug therapy and those who did not receive any additional information except standard medication counseling.

***Study objectives***

The aim of the SMAAP-AF study is to investigate adherence to the DOAC edoxaban and apixaban in Stage 1 and whether the educational program for medication modifies medication adherence in patients with NVAF treated with DOACs in Stage 2, with differences in the number of required daily doses measured using the electronic monitoring device. The primary endpoint is medication adherence, which will be compared between patients who receive the educational program and those who do not receive it in Stage 2. The secondary endpoint is the status of medication adherence, which will be compared between patients treated with edoxaban and those treated with apixaban in Stage 1. The following items through Stage 1 and Stage 2 will also be investigated: 1) the Morisky Medication Adherence Scale-8 (MMAS-8) [1]; 2) major bleeding; 3) major cardiovascular events (ischemic stroke, systemic embolism) and 4) other adverse events.

***Assessment***

The status of medication adherence will be measured using the electronic monitoring device “Your Manager” (DNP, Tokyo, Japan) to objectively evaluate medication adherence. “Your Manager” is a card-type press-through pack (PTP) electronic device [2] that records the date and time when each tablet in the PTP is opened. Data are recorded in a comma separated value (CSV) format to be captured electrically.

In addition, to perform an evaluation using an adherence questionnaire, the Japanese version of the MMAS-8, which consists of eight questions, will be used. The total score is calculated by adding 0 points for “Yes” and 1 point for “No.” The higher the total score, the higher the adherence. [1]

***Educational program for medication***

In Stage 1, patients who are enrolled in this study will be provided with standard medication counseling by pharmacists at the time of drug delivery. In addition to standard medication counseling, patients who are allocated to the educational program group will be given specific instructions regarding how to take their medication; these instructions will be generated by the Patient Education Advisor for this study as a special program.

The proposed educational intervention program involves motivational interviewing (MI). This conversation method is based on the four concepts of Partnership, Acceptance, Compassion, and Evocation (PACE). [3] Through extensive MI, patients will be ranked according to their “motivation” and “self-determination” toward keeping up with the guidelines of their anticoagulation therapy. The MI process utilizes four skills, represented by the acronym OARS, for the advisor to engage with the patient—asking the patients about their opinions and feelings (asking Open question); prompting the patients to identify and mention their own strengths, efforts, and positive behavior in the conversation (Affirming); posing meaningful questions to the patients based on decoding their said, or sometimes unsaid, remarks (Reflective listening); and summarizing the patients’ stories and thoughts (Summarizing). In the case of this work, PACE and OARS will be effectively leveraged with regards to leading the patients in maintaining or improving their adherence to their therapy (change talk). To ensure uniform implementation of PACE and OARS, the pharmacist in charge will ask the patient several questions concerning their situation according to the MI sheet (e.g. “How have you been?”, “Do you have any trouble taking your medication?”, “How is the effect of the medication?”, “Is there anything you want to do?”). During this process, the topic is decided, and the patients’ answers are specified and clarified. The Education Intervention Program is conducted by certified pharmacists who have received training on patient interviews, conducted by the advisor of the Medication Education Program for this study.

***Sample size estimation***

According to a previous report [4], the nonadherence rate (<80% rate of medication rate) was 34.8% (363/1042) among patients who did not receive any specific drug education and 19.7% (63/320) among those who received drug education in person or by telephone. In that study, the relative risk adjusted by sex, age, and race was 1.26, whereas the crude odds ratio for the noncompliance rate was 0.46. If the number of patients completing the intervention (Stage 2) is 320 patients in total, the power of detection is 86.1% when the significance level is 5%. Therefore, the planned number of patients is considered sufficient for this study’s purpose.

References

1. Morisky DE, Ang A, Krousel-Wood M, Ward HJ. Predictive validity of a medication adherence measure in an outpatient setting. J Clin Hypertens (Greenwich). 2008; 10:348-354.
2. Sadamoto K, Takamori H, Sadamoto T, Kubota K. Impact of push-through-packages with electronic devices for accurate drug taking. Journal of Scientific and Innovative Research. 2014; 3:288-294.
3. Miller WR and Rollnick S. Motivational Interviewing: Helping people Change. 3rd ed. New York; Guilford Press; 2012.
4. Shore S, Ho PM, Lambert-Kerzner A, Glorioso TJ, Carey EP, Cunningham F, et al. Site-level variation in and practices associated with dabigatran adherence. JAMA 2015; 313:1443-1450.
