# Supplementary material for: Electronic monitoring of adherence to once‐daily and twice‐daily direct oral anticoagulants in patients with atrial fibrillation: Baseline data from the SMAAP‐AF trial
Source: J Arrhythm. 2021 Mar 30;37(3):616–25. doi: 10.1002/joa3.12532 (PMC8207342; doi:10.1002/joa3.12532)
Supplement: Supplementary file 2 — Table S1 [file JOA3-37-616-s002.docx]

Table S1. In- and exclusion criteria per SMAAP-AF trial

| Inclusion criteria | Exclusion criteria |
| --- | --- |
| 1) Patients aged ≥20 years.  2) Outpatients with NVAF.  3) Patients taking edoxaban or apixaban for at least 4 weeks prior to enrollment in this study.  4) Patients from whom written informed consent was obtained based on the patient’s own free will and with sufficient understanding after receiving sufficient explanation for participation in this study. | 1) Patients participating in or planning to participate in other intervention studies or trials.  2) Patients who may have difficulty maintaining adherence.  3) Patients whose medications are managed by a person other than themselves.  4) Patients who are or will be hospitalized during the study period.  *If the patient is hospitalized during the study, the study will be suspended for that patient due to prioritizing treatment for the condition requiring hospitalization. The study can be resumed in accordance with a doctor’s decision once the patient returns for outpatient visits. The Case Evaluation Committee will discuss how to deal with the hospitalization period.  5) Pregnant women or patients who may be pregnant.  6) Patients judged unsuitable for this study by an investigator or a sub-investigator. |

NVAF, nonvalvular atrial fibrillation
